# Nomogram to Predict Long-Term Overall Survival and Cancer-Specific Survival of Radiotherapy Patients with Nasopharyngeal Carcinoma

**DOI:** 10.1155/2023/7126881

**Published:** 2023-01-17

**Authors:** Zhiru Li, Chao Li, Dong Yang, Ziyan Zhou, Min Kang

**Affiliations:** ^1^Department of Radiation Oncology, Sichuan Provincial People's Hospital Qionglai Medical Center Hospital, Chengdu, Sichuan, China; ^2^Department of Radiation Oncology, The First Affiliated Hospital of Guangxi Medical University, Nanning, Guangxi, China; ^3^Guangxi Tumor Radiation Therapy Clinical Medical Research Center, Nanning, Guangxi, China; ^4^Department of Obstetrics and Gynecology, Sichuan Provincial People's Hospital Qionglai Medical Center Hospital, Chengdu, Sichuan, China

## Abstract

**Objective:**

To establish and validate a nomogram to predict the overall survival (OS) and cancer-specific survival (CSS) in patients with nasopharyngeal carcinoma (NPC) receiving radiotherapy by integrating multiple independent prognostic factors.

**Materials and Methods:**

Data from 5663 patients with NPC who received definite radiotherapy between 2004 and 2018 were included and divided into training and validation cohorts. Univariate and multivariate Cox regression analyses were performed to determine the independent prognostic factors of patients with NPC after radiotherapy. Thereafter, the predictive accuracy of the nomogram model was evaluated.

**Results:**

Age, race, marital status, pathological type, tumor size, T stage, N stage, M stage, American Joint Committee on Cancer stage, and chemotherapy were independent factors affecting the prognosis of patients with NPC receiving radiotherapy. Nomograms with a concordance index of 0.726 (95% confidence interval (CI): 0.675–0.777) and 0.732 (95% CI: 0.680–0.785) were able to predict OS and CSS, respectively. The area under the curve showed excellent predictive performance. Additionally, the calibration curve indicated that the predicted survival rate was consistent with the actual survival rate, and the decision curve indicated its clinical value. The established risk stratification system was able to accurately stratify patients receiving radiotherapy for NPC into three risk subgroups with significant differences in prognosis (*P* < 0.05).

**Conclusions:**

The constructed nomogram had good prognostic performance and could be used as an effective tool to evaluate the prognosis of patients with NPC after radiotherapy. This nomogram could be further used to guide clinical decisions and personalized treatment plans.

## 1. Introduction

Nasopharyngeal carcinoma (NPC) is one of the most common head and neck malignancies, which occurs mainly in Guangdong, Hainan, Hunan, and other regions in China [1, 2]. NPC is sensitive to radiotherapy, which is the first choice for radical treatment, as alone it can achieve a good prognosis for early lesions [3, 4]. However, the initial symptoms of NPC are often unnoticed, and regional lymph nodes and/or blood vessel metastasis can occur easily. The majority of NPC patients are already in the locally advanced stage when they are first admitted to a hospital, and the efficacy of radiotherapy alone is not favorable. The 5-year survival rate for NPC patients has been reported to range between 50% and 60%, with the main reasons for treatment failure being local recurrence and distant metastasis [5–7]. With the development of imaging and intensity-modulated radiotherapy technology and the application of radiochemical combined therapies, the efficacy of NPC treatment has significantly improved, with the 5-year local control and survival rates being approximately 90% and 80%, respectively [8]. The factors influencing the prognosis of patients with NPC after radiotherapy mainly include the general health of patients, disease factors, and treatment factors [5, 7]. Individual prognostic prediction is important for NPC patients undergoing radiotherapy. Traditional staging systems, such as the American Joint Committee on Cancer (AJCC) staging classification [9], are generally applicable to patient groups rather than to specific individuals. Clinical guidelines for identifying clinical risk are usually based on the tumor node metastases (TNM) staging at diagnosis. Additionally, TNM staging only considers tumor size, lymph node, and distant metastases, regardless of age, sex, tumor grade, treatment, and other prognostic factors [10, 11]. Therefore, there is an urgent need to develop a more accurate analysis tool to predict the individual prognosis of NPC patients after radiotherapy and to accurately stratify patients with different risk profiles [12, 13].

In recent years, various studies have used nomograms to construct prediction models as a tool for oncology and medical prognosis evaluation. Nomograms can generate individual predictions by integrating multiple prognostic and determining variables for personalized patient identification and stratification [13, 14]. The Surveillance, Epidemiology, and End Results (SEER) database is the largest publicly available tumor registry database in the United States (US), which includes demographic, clinicopathological, survival, and clinical data of patients with various types of tumors, covering approximately 30% of the US population [15, 16]. The SEER database collates data from 18 cancer registry databases [15]. The aim of this study was to use the SEER database to screen patients with NPC and randomly divide them into training and validation cohorts to construct a new risk stratification system based on the individual prediction scores of the nomogram. Additionally, it is aimed at predicting overall survival (OS) and cancer-specific survival (CSS) and at evaluating the performance of the statistical prediction model. In doing so, we hoped to identify high-risk patients and guide clinical treatment decisions for NPC.

## 2. Materials and Methods

### 2.1. Data Sources and Study Populations

This was a retrospective study where information on patients diagnosed with NPC between 2004 and 2018 was obtained from the SEER database using the SEER^∗^Stat software (version 8.3.9, http://www.seer.cancer.gov/seerstat). The inclusion criteria were as follows: (1) patients were diagnosed between 2004 and 2018; (2) their tumors were staged according to the 6^th^, 7^th^, and 8^th^ versions of AJCC (2004–2009, 2010–2017, and 2018, respectively) and were available in the SEER database; (3) they had newly diagnosed NPC without a history of surgery; (4) they had received definite radiotherapy; and (5) the International Classification of Diseases for Oncology, 3^rd^ Edition (ICD-O-3), was used to classify tumors according to histological types determined by the World Health Organization (WHO) classification scheme. Squamous cell carcinoma (SCC) (ICD-O-3 codes 8070 and 8071) represents the histological subtypes of keratinizing squamous cell carcinoma (KSCC), while nonkeratinizing carcinoma (NKC) (ICD-O-3 codes 8072 and 8073) represents the histological subtypes of differentiated NKC. Undifferentiated, anaplastic, and lymphoepithelial carcinomas (ICD-O-3 codes 8020, 8021, and 8082) represent undifferentiated NKC histologic subtypes. Carcinoma that was not specified (ICD-O-3 code 8010) was assigned to form an NOS histologic subtype. For such cases, the causes of death and survival time were known.

Patients were excluded if (1) the diagnostic method was unknown; (2) their race and/or marital status was unknown; (3) their cause of death was unknown; (4) they had an unknown or incomplete TNM staging and/or an unknown metastasis; (5) their radiotherapy status was unknown; (6) their available follow-up time was less than 1 month; and (7) they received nonbeam radiation. Beam radiotherapy was defined as external beam radiation using gamma rays generated from a cobalt-60 source or high-energy photons generated from a linear accelerator. Nonbeam radiation was defined as any type of brachytherapy, unsealed source, intraoperative radiation not otherwise specified, or radiation not otherwise specified. This group of patients was excluded to reduce potential selection bias caused by different radiotherapy modalities.

### 2.2. Patient Data Collection

**Figure 1:**
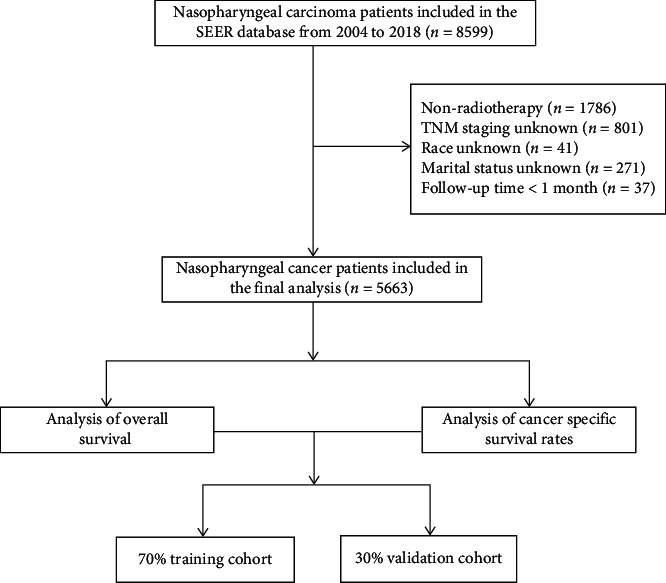
Analysis flowchart.

Data from 8599 patients with NPC was collected from the SEER database. The data processing flowchart is shown in Figure 1. Overall, 5663 patients with NPC were included in this study. All patients were randomly assigned to training (*N* = 3967) and validation (*N* = 1696) cohorts using a 7 : 3 ratio. The variables included for each patient were age, sex, race, year of diagnosis, marital status, pathological type, histological grade, tumor size, TNM stage, AJCC stage, chemotherapy record, diagnostic confirmation record, cause-specific death classification, follow-up time, and survival status. The main endpoints for this study were OS and CSS. OS was defined as the time between the date of NPC diagnosis and the date of death from any cause or the last follow-up date. CSS was defined as the time from the date of diagnosis to death attributable to NPC.

### 2.3. Statistical Methods

Statistical analysis was conducted using SPSS version 25.0 and R version 4.1.2. The R function “createDataPartition” was used to divide the patients into training and validation cohorts using a 7 : 3 ratio to ensure randomization. The training cohort was used to filter variables and build prediction models. Variables that were significant in the univariate Cox regression model were included in the multivariate Cox regression model, and multivariate proportional hazard models (hazard ratios and 95% confidence intervals (CI)) were used to identify independent prognostic factors associated with OS and CSS. The prognostic nomogram was constructed based on the multivariate analysis of the training cohort, and it was used to predict the 3- and 5-year OS and CSS by representing the total points for each variable [17]. Individual scores were read based on each clinicopathological indicator for each patient, and the scores were added together to obtain a total score. The lower the total score, the higher the 3- and 5-year survival rates. The accuracy of the prognostic model was evaluated by calculating the concordance index (C-index) and the receiver operating characteristic (ROC) curve. The time-dependent area under the curve (AUC) was not only used to evaluate the predictive power of the nomogram but was also used to compare the predictive ability of the AJCC stage. The calibration curve was used to compare the consistency of the predicted OS and CSS with the actual survival rate, and the decision curve was used to evaluate the clinical applicability of this nomogram by quantifying the net improvement benefit at different threshold probabilities. The risk stratification system was established based on the scores of each patient in the training cohort, and the optimal cut-off values of continuous variables were determined using the X-tile software. The patients were divided into three subgroups, namely, low-, medium-, and high-risk groups. Each risk subgroup represented a different prognosis. The Kaplan-Meier survival analysis was used to generate survival curves for the different risk groups, and log-rank tests were used for comparison between the groups. *P* < 0.05 was considered statistically significant.

## 3. Results

### 3.1. General Information

In total, 5663 screened patients with NPC had a median follow-up of 44 months. Overall, 3967 and 1696 patients were randomly divided into the training and validation cohorts, of whom 1556 and 673 died, respectively. Clinical and demographic information for the two groups is shown in Table 1; there were no significant differences between the two groups (*P* > 0.05).

### 3.2. Independent Prognostic Factors in the Training Cohort

Univariate Cox regression analysis showed that there were significant differences in OS and CSS for the evaluated prognostic factors (*P* < 0.05) with the exception of sex (Table 2). In the multivariate Cox regression analysis of OS and CSS, age, race, marital status, pathological type, tumor size, T stage, N stage, M stage, AJCC stage, and chemotherapy were independent factors affecting OS and CSS in NPC patients undergoing radiotherapy (all *P* < 0.05) (Table 3).

### 3.3. Construction and Validation of a Nomogram for Predicting the Total Survival Rate

**Figure 2:**
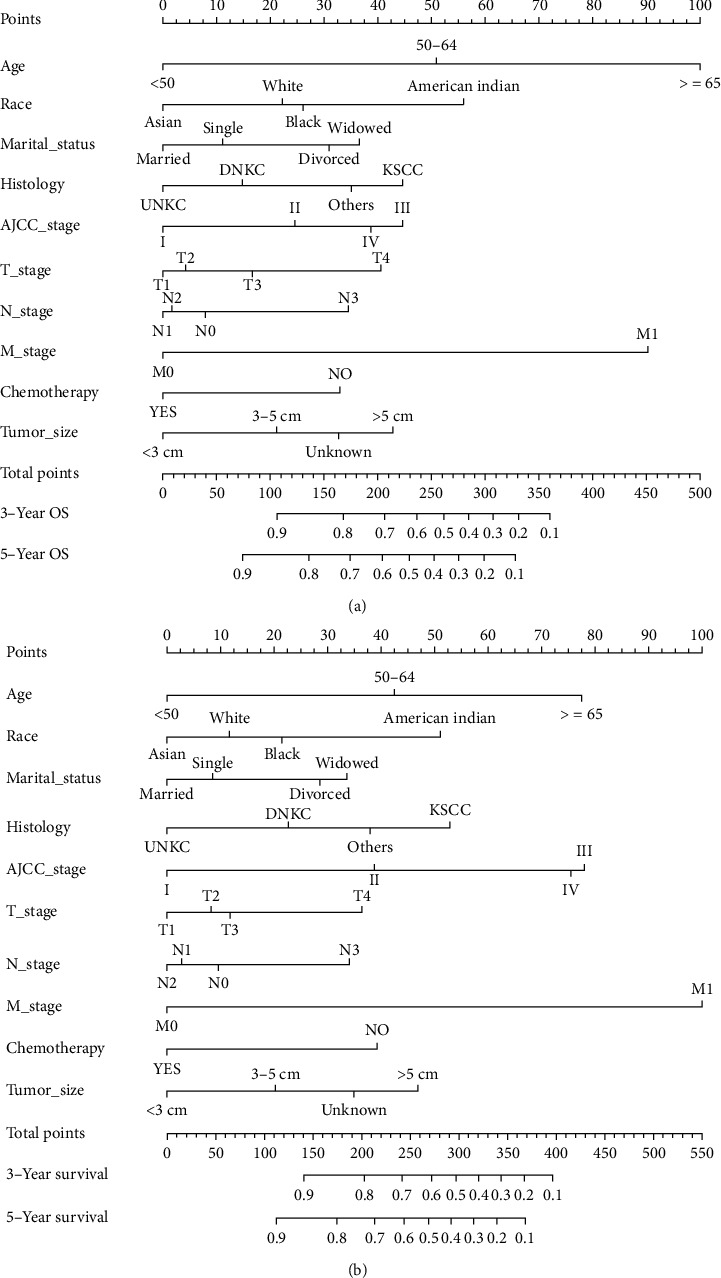
Nomogram to predict overall survival (a) and cancer-specific survival (b) in radiotherapy patients with nasopharyngeal carcinoma undergoing radiotherapy.

**Figure 3:**
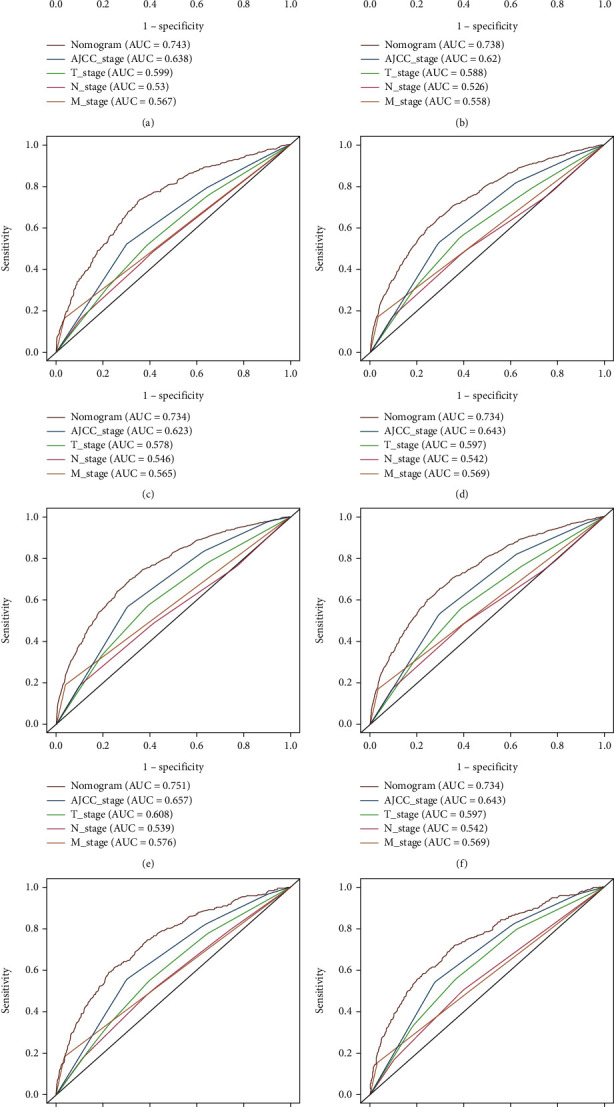
Receiver operating characteristic curves of the nomogram to predict overall survival (OS) and cancer-specific survival (CSS). (a) 3-year OS in training cohort; (b) 5-year OS in training cohort; (c) 3-year OS in validation cohort; (d) 5-year OS in validation cohort; (e) 3-year CSS in training cohort; (f) 5-year CSS in training cohort; (g) 3-year CSS in validation cohort; (h) 5-year CSS in validation cohort. Abbreviations: AUC: area under the curve; ROC: receiver operating characteristic.

**Figure 4:**
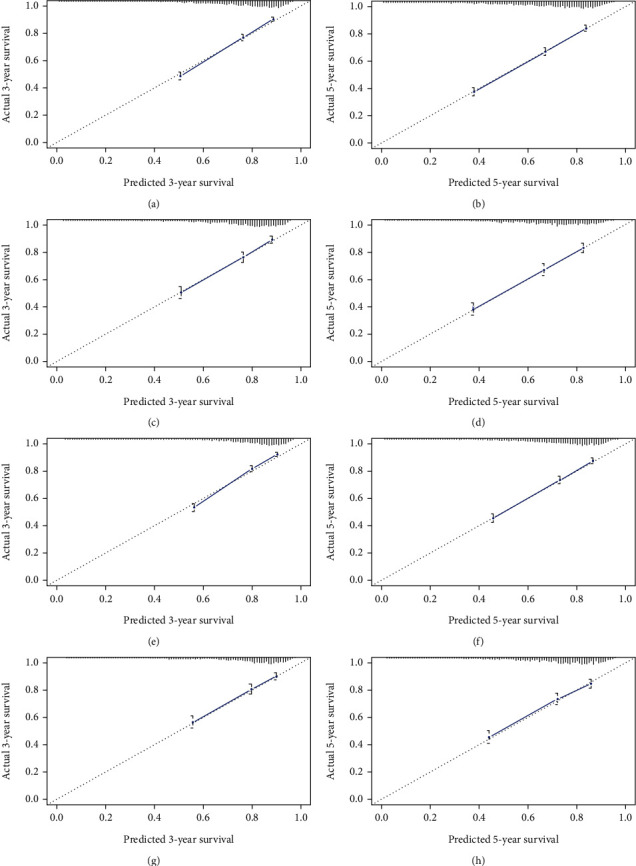
Calibration curves of the nomogram to predict overall survival (OS) and cancer-specific survival (CSS). (a) 3-year OS in training cohort; (b) 5-year OS in training cohort; (c) 3-year OS in validation cohort; (d) 5-year OS in validation cohort; (e) 3-year CSS in training cohort; (f) 5-year CSS in training cohort; (g) 3-year CSS in validation cohort; (h) 5-year CSS in validation cohort.

**Figure 5:**
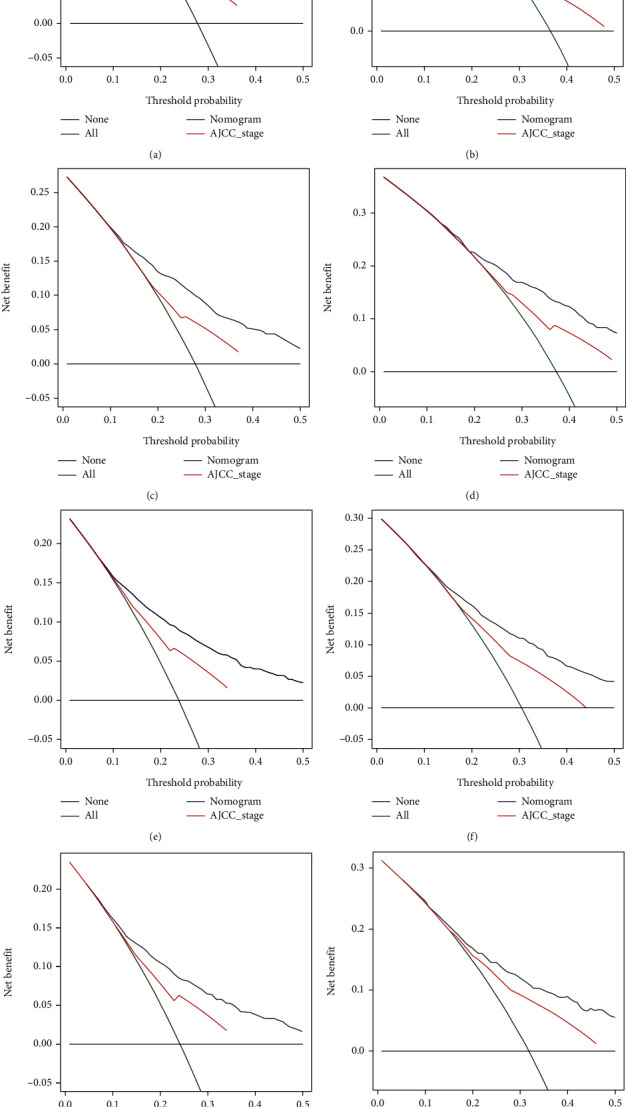
Decision curves for nomogram and American Joint Committee on Cancer staging systems. (a) Decision curves for 3-year OS prediction in training cohort; (b) Decision curves for 5-year OS prediction in training cohort; (c) decision curves for 3-year OS prediction in validation cohort; (d) decision curves for 5-year OS prediction in validation cohort; (e) decision curves for 3-year CSS prediction in training cohort; (f) decision curves for 5-year CSS prediction in training cohort; (g) decision curves for 3-year CSS prediction in validation cohort; (h) decision curves for 5-year CSS prediction in validation cohort. Abbreviations: OS: overall survival; CSS: cancer-specific survival.

Based on independent prognostic factors determined in the multivariate Cox regression analysis, a nomogram was constructed to predict 3- and 5-year OS and CSS for the training cohort (Figure 2). Age, race, and M stage had greater efficacy in the construction of the prediction model. The scores of each variable were added together to obtain 3- and 5-year OS and CSS. The C-index for OS was higher than that of the AJCC staging system (0.726 vs. 0.603), and that of the CSS was higher than that of OS (0.732 vs. 0.726). The AUC of the 3- and 5-year survival rates in the OS prediction model of the training cohort were 0.743 and 0.738, respectively, and those in the CSS prediction model were 0.751 and 0.734, respectively. The curve in Figure 3 shows that the AUC value for the OS/CSS nomogram was higher than that of the AJCC stage and separate T, N, and M stages, and the predictive ability of the nomogram was better. Additionally, the calibration curves show that the nomogram's prediction of the 3- and 5-year OS and CSS was in agreement with the actual survival rates (Figure 4). The decision curve for the nomogram and AJCC staging prediction model is shown in Figure 5. The net benefit was higher than that of the AJCC model in OS and CSS prediction at 3 and 5 years.

**Figure 6:**
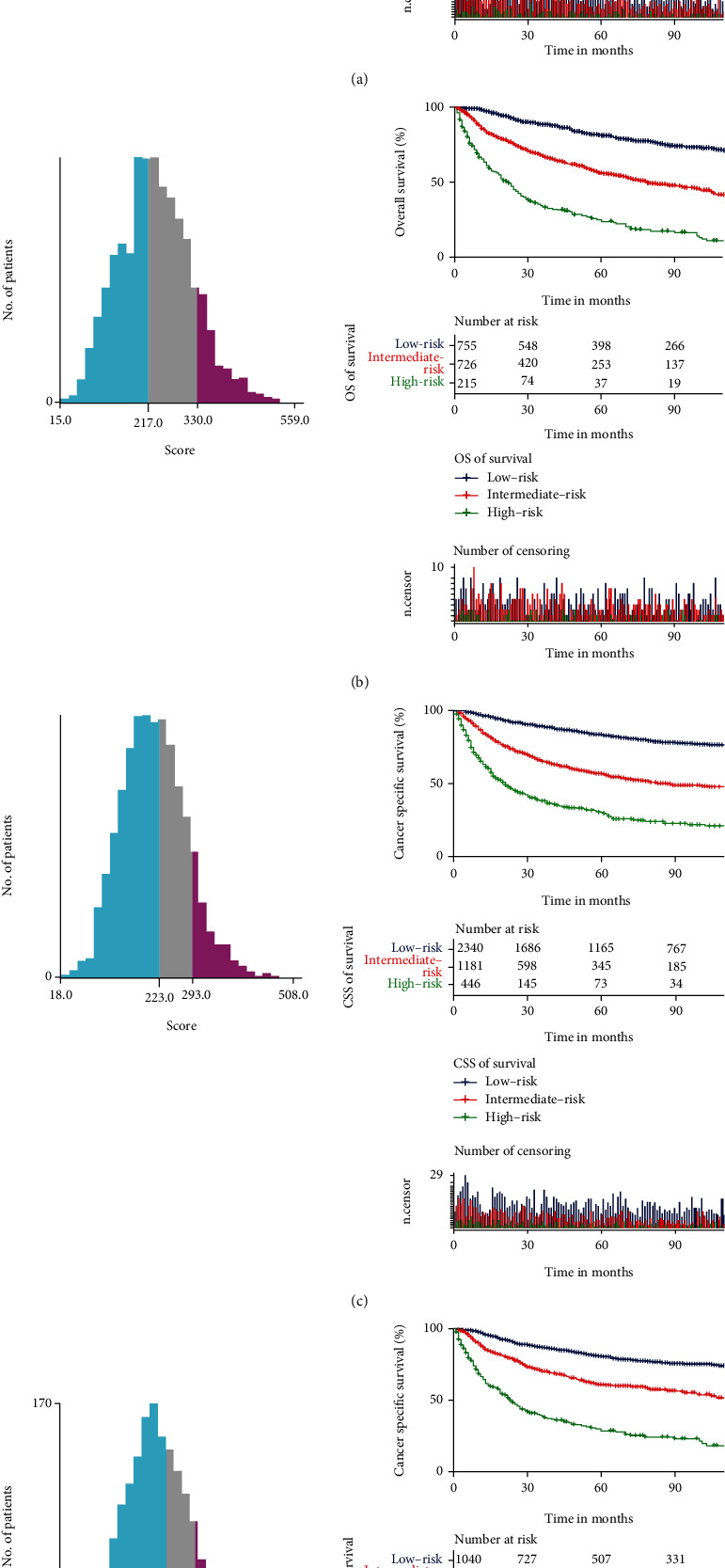
The Kaplan-Meier analysis of overall survival and cancer-specific survival in the three groups in the risk stratification system. The Kaplan-Meier analysis of overall survival for patients stratified by the risk stratification system in the training cohort (a) and validation cohort (b). The Kaplan-Meier analysis of cancer-specific survival for patients stratified by the risk stratification system in the training cohort (c) and validation cohort (d).

The Kaplan-Meier survival curve for the prognostic risk stratification system found that OS and CSS in the three subgroups were accurately separated by the system, and there was a significant difference in survival among the three groups (*P* < 0.05) (Figure 6).

## 4. Discussion

Radiotherapy is still the cornerstone of NPC treatment [18, 19]. In the past two decades, there have been significant changes in the standard of care for NPC, including the use of the AJCC/Union for International Cancer Control (UICC) staging system [20, 21], and the use of magnetic resonance imaging (MRI) as standard radiologic staging for local disease. Cisplatin chemotherapy is used in combination with radiotherapy for patients with locally advanced disease, and intensity-modulated radiotherapy (IMRT) is used as standard radiotherapy [22].

The AJCC/UICC TNM staging system is the most widely accepted universal language for describing tumors, is the most common staging system for NPC, and is an important basis for guiding clinical activity in judging prognosis [23–25]. This staging system is continually updated, with the latest edition (8^th^ edition) relying on improved local control of IMRT, as well as more detailed MRI preprocessing imaging, which results in a more consistent definition of T2 (with T4) and N3 diseases [26]. However, AJCC stage is not the only factor affecting the prognosis of NPC, and the survival time of NPC patients with the same stage varies. A growing number of studies have incorporated clinicopathologic features and clinical treatment information, including demographic statistical variables, Epstein-Barr virus- (EBV-) DNA, hematological markers of inflammation, and gross tumor volume, to further improve the prognostic accuracy [27].

NPC is sensitive to radiotherapy and chemotherapy. The combination of chemotherapy and radiotherapy is considered a breakthrough in the treatment of locally advanced NPC. A number of randomized trials have shown relatively consistent results, with chemotherapy plus radiotherapy having better survival correlates [28, 29]. Au et al. demonstrated that the use of chemotherapy (neoadjuvant plus concurrent, concurrent, and adjuvant plus concurrent) was an independent favorable prognostic factor for OS and PFS compared with IMRT alone, confirming the benefit of chemotherapy for IMRT [22]. The presence of plasma EBV DNA in patients with NPC is associated with apoptosis of tumor tissue and has the same polymorphism as the primary site, and plasma EBV DNA is strongly associated with tumor grade. Consequently, it has become the most accurate biomarker for screening, diagnosis, monitoring response to treatment, monitoring recurrence, and prediction of NPC in endemic areas [30, 31]. Two meta-analyses have also shown that pretreatment plasma EBV DNA titers are an important prognostic factor in NPC [32, 33]. In 1978, the WHO classified NPC histologically into three categories: squamous cell carcinoma (WHO-I), NKC (WHO-II), and undifferentiated carcinoma (WHO-III). This WHO classification was revised in 1991, and NPCs were classified as KSCC and NKC. NKC was then further subdivided into differentiated and undifferentiated. The third edition of the WHO classification of NPC added basal cell squamous cell carcinoma as one of the histological subtypes in 2003 [34]. Histological subtypes of NPC have a distinct geographic and ethnic distribution in both endemic and nonendemic areas of NPC, and in endemic areas, such as southern China, more than 95% of NPC patients have NKC; however, KSCC is more common in nonendemic areas [22, 27]. Pan et al. showed that histological subtypes determine long-term survival outcomes in NPC patients. Long-term survival outcomes are worse in patients with KSCC, whereas survival is higher in patients with NKC [35].

In this study, age, race, marital status, pathologic type, tumor size, T stage, N stage, M stage, AJCC stage, and chemotherapy were identified as independent prognostic variables for OS. The nomogram is based on multivariate regression analysis, which calculates the individual end event forecast value using the individual score and end event occurrence probability function transformation. Previous studies have shown that complex regression equations can be transformed into visual graphs to make the results of the prediction model more readable and to facilitate the evaluation of patients [36, 37]. Traditionally, AJCC staging, which is closely related to OS, has been the first choice for the prediction of NPC prognosis. However, the prognosis of patients with the same staging can differ. This heterogeneity occurs because AJCC staging does not consider other factors such as age, pathology, adjuvant therapy, marital status, and/or race [38, 39]. In predicting the prognosis of NPC, the nomogram reduces the diversity due to different treatments and sociodemographic and treatment statuses. However, there is still a lack of a complete and effective nomogram prediction model for NPC patients undergoing radiotherapy, and only a few studies have applied it to visual prediction models [40, 41]. Moreover, the stability and generality of the alignment chart may be reduced as a result of the smaller sample size.

Our study developed a 3- and 5-year OS and CSS nomogram for NPC patients based on 5663 samples obtained from the SEER database. Among them, M staging, age, and race contributed the most substantially to the OS nomogram, while M staging, age, and AJCC staging contributed the most substantially to the CSS nomogram. Although we used different versions of AJCC staging system, it was helpful to analyze the results and showed a good comparability. By comparing the C-index of the nomogram and AJCC stages, the C-index of the nomogram for OS and CSS prediction was found to be higher than that of AJCC in the training and validation cohorts. These findings suggest that this model has a good recognition ability, and the C-index is better than the AJCC staging prediction model. The correction curve showed that the predicted probability of the nomogram was in agreement with the actual probability calculated using the Kaplan-Meier analysis, and the 3- and 5-year survival rates were in good agreement. These results highlight that this nomogram could be used in clinical practice with less deviation and better accuracy. Decision curve analysis combines benefits and harms to measure the net benefits of an approach or predictive model. Compared with the ROC curve, the decision curve considers the clinical application value, which is an important indicator to evaluate whether patients can benefit from it. However, only a few papers have applied it to the survival prediction for NPC. In our study, we calculated not only the net benefit of the nomogram but also evaluated the AJCC staging prediction model and found that the net benefit of the nomogram was higher than that of the AJCC staging prediction model, indicating that this nomogram is feasible for clinical use.

Additionally, we established a prognostic nomogram risk stratification system to accurately classify patients into three risk subgroups and identify high-risk subgroups that may require more intensive treatment. This provided guidance for patient counseling and clinical risk management. For high-risk patients, more psychological and emotional care may also be needed. Additionally, they may be encouraged to participate in clinical trials evaluating new drugs, such as immunotherapy and targeted drugs. The use of this model for stratification helped to reduce heterogeneity among the different treatment groups and thus should be considered for future use.

This study has some limitations which merit mentioning. First, it was a retrospective study, in which patient selection was based on rigorous inclusion and exclusion criteria; thus, potential selection bias may have occurred. Second, the SEER database does not specify procedures, operators, and other factors, which may induce bias due to the different experience levels of the operators and pathologists. Third, because of the limited number of variables collected in the SEER database, some important variables were not available, such as Epstein-Barr virus (EBV) infection, complications, detailed chemotherapy regimens, chemotherapy course and duration, AJCC integration, and staging information based on MRI images. Fourth, SEER does not mention tumor location (primary or metastatic) or dose and mode of radiotherapy, and it does not specify the specific radiotherapy technique, making the study of the survival benefit of patients treated with radiotherapy somewhat limited. Finally, in the absence of external validation, analysis of US population-based data is not necessarily generalizable to the global population, especially to patients with NPC in endemic areas. Therefore, to improve the predictive value of the model, it will be important to consider integrating multicenter Chinese and foreign databases in the future.

## 5. Conclusions

Based on clinical features and treatment-related information evaluated, our study established a useful nomogram for the prediction of 3- and 5-year OS and CSS in patients with NPC receiving radiotherapy. The results showed that our nomogram was superior to the AJCC staging prediction model and was successful in predicting individual survival. Further external validation using prospective data should be conducted to verify these findings. Nonetheless, this nomogram could be used in the future to assist clinicians in devising the best treatment plans for patients with NPC.

## Figures and Tables

**Table 1:** Baseline characteristics of patients with nasopharyngeal carcinoma in the training cohort and validation cohort (*N*, %).

Characteristics	Total (cases, %)	Modeling group (cases, %)	Validation group (cases, %)	*P* value
Total	5663	3967	1696	
Age group (years)				
<50	1941 (34.28)	1362 (34.33)	579 (34.14)	0.92
50-64	2335 (41.23)	1637 (41.27)	698 (41.15)	
≥65	1387 (24.49)	968 (24.40)	419 (24.71)	
Sex				
Male	4008 (70.78)	2823 (71.16)	1185 (69.87)	0.619
Female	1655 (29.22)	1144 (11.87)	511 (30.13)	
Race				
Black	685 (12.10)	471 (11.87)	214 (12.62)	0.980
White	2665 (47.06)	1873 (47.21)	792 (46.69)	
Asian/Pacific islander	2233 (39.43)	1564 (39.43)	669 (39.45)	
Indian/Alaska native	80 (1.41)	59 (1.49)	21 (1.24)	
Marital status				
Single	1242 (21.93)	888 (22.38)	354 (20.87)	0.893
Married	3540 (62.51)	2473 (62.34)	1067(62.91)	
Divorced	521 (9.20)	362 (9.13)	159 (9.38)	
Windowed/separated	360 (6.36)	244 (6.15)	116 (6.84)	
Histology				
KSCC	1887 (33.32)	1329 (33.51)	558 (32.90)	0.182
DNKC	1458 (25.75)	982 (24.75)	476 (28.07)	
UNKC	944 (16.67)	661 (16.66)	283 (16.68)	
Others	1374 (24.26)	995 (25.08)	379 (22.35)	
Grade				
Well	92 (1.62)	63 (1.59)	29 (1.71)	0.762
Moderate	476 (8.41)	317 (7.99)	159 (9.38)	
Poor	1626 (28.71)	1158 (29.19)	468 (27.59)	
Undifferentiated	1571 (27.74)	1086 (27.38)	485 (28.6)	
Unknown	1898 (33.52)	1343 (33.85)	555 (32.72)	
Tumor size (cm)				
<3	1085 (19.16)	754 (19.01)	331 (19.52)	0.815
3-5	1661 (29.33)	1173 (29.57)	488 (28.77)	
>5	735 (12.98)	497 (12.53)	238 (14.03)	
Unknown	2182 (38.53)	1543 (38.89)	639 (37.68)	
AJCC stage				
I	462 (8.16)	317 (7.99)	145 (8.55)	0.997
II	1349 (23.82)	948 (23.89)	401 (23.64)	
III	1789 (31.59)	1251 (31.54)	538 (31.72)	
IV	2063 (36.43)	1451 (36.58)	612 (36.09)	
T stage				
T1	1817 (32.09)	1273 (32.09)	544 (32.07)	0.997
T2	1387 (24.49)	963 (24.28)	424 (25.00)	
T3	1168 (20.63)	827 (20.85)	341 (20.11)	
T4	1291 (22.79)	904 (22.78)	387 (22.82)	
N stage				
N0	1372 (24.23)	959 (24.17)	413 (24.35)	0.983
N1	1869 (30.78)	1313 (33.10)	556 (32.78)	
N2	1743 (30.78)	1230 (31.01)	513 (30.25)	
N3	679 (11.99)	465 (11.72)	214 (12.62)	
M stage				
M0	5245 (92.62)	3667 (92.44)	1578 (93.04)	0.728
M1	418 (7.38)	300 (7.56)	118 (6.96)	
Chemotherapy				
Yes	4918 (86.84)	3448 (86.92)	1470 (86.67)	0.970
No/unknown	745 (13.16)	519 (13.08)	226 (13.33)	

**Table 2:** Univariate Cox regression analysis of overall survival and cancer-specific survival in nasopharyngeal carcinoma in the training cohort.

Variables	Overall survival			Cancer-specific survival		
	HR	95% CI	*P*	HR	95% CI	*P*
Total						
Age group (years)						
<50	Reference			Reference		
50-64	1.854	1.628-2.112	<0.001^∗^	1.639	1.420-1.891	<0.001^∗^
≥65	3.419	2.989-3.912	<0.001^∗^	2.490	2.136-2.903	<0.001^∗^
Sex						
Male	Reference			Reference		
Female	0.884	0.791-0.9883	0.03	0.882	0.775-1.002	0.054
Race						
Black	Reference			Reference		
White	1.031	0.886-1.201	0.693	0.924	0.777-1.099	0.372
Asian/Pacific islander	0.601	0.509-0.707	<0.001^∗^	0.631	0.525-0.759	<0.001^∗^
Indian/Alaska native	1.455	1.017-2.083	0.040	1.412	0.939-2.122	0.097
Marital status						
Single	Reference			Reference		
Married	0.971	0.855-1.104	0.655	0.925	0.801-1.069	0.292
Divorced	1.624	1.357-1.945	<0.001^∗^	1.493	1.213-1.837	<0.001^∗^
Windowed/separated	2.486	2.058-3.004	<0.001^∗^	2.113	1.693-2.636	<0.001^∗^
Histology						
KSCC	Reference			Reference		
DNKC	0.599	0.523-0.686	<0.001^∗^	0.619	1.616-0.722	<0.001^∗^
UNKC	0.446	0.379-0.522	<0.001^∗^	0.429	2.328-0.518	<0.001^∗^
Others	0.724	0.639-0.820	<0.001^∗^	0.707	1.414-0.817	<0.001^∗^
Grade						
Well	Reference			Reference		
Moderate	1.106	0.774-1.580	0.581	1.135	0.747-1.726	0.553
Poor	0.744	0.531-1.043	0.086	0.795	0.535-1.182	0.258
Undifferentiated	0.463	0.329-0.653	<0.001^∗^	0.478	0.319-0.715	<0.001^∗^
Unknown	0.667	0.475-0.936	0.019	0.699	0.469-1.042	0.079
Tumor size (cm)						
<3	Reference			Reference		
3-5	1.348	1.147-1.585	<0.001^∗^	1.377	1.137-1.667	<0.001^∗^
>5	2.089	1.738-2.510	<0.001^∗^	2.387	1.933-2.946	<0.001^∗^
Unknown	1.521	1.308-1.768	<0.001^∗^	1.634	1.368-1.953	<0.001^∗^
AJCC stage						
I	Reference			Reference		
II	1.038	0.827-1.303	0.748	1.259	0.924-1.716	0.144
III	1.289	1.036-1.603	0.023	1.857	1.383-2.494	<0.001^∗^
IV	2.268	1.838-2.799	<0.001^∗^	3.551	2.668-4.726	<0.001^∗^
T stage						
T1	Reference			Reference		
T2	1.117	0.968-1.288	0.129	1.217	1.028-1.441	0.023
T3	1.555	1.351-1.792	<0.001^∗^	1.716	1.453-2.027	<0.001^∗^
T4	1.974	1.726-2.258	<0.001^∗^	2.303	1.970-2.692	<0.001^∗^
N stage						
N0	Reference			Reference		
N1	0.76	0.666-0.867	<0.001^∗^	0.803	0.686-0.939	0.006
N2	0.876	0.767-0.999	0.049	0.997	0.855-1.163	0.970
N3	1.284	1.093-1.509	0.002	1.536	1.280-1.844	<0.001^∗^
M stage						
M0	Reference			Reference		
M1	3.182	2.756-3.676	<0.001^∗^	3.905	3.353-4.549	<0.001^∗^
Chemotherapy						
Yes	Reference			Reference		
No/unknown	1.312	1.144-1.505	<0.001^∗^	1.207	1.026-1.421	<0.001^∗^

**Table 3:** Multivariate Cox regression analysis of overall survival and cancer-specific survival in nasopharyngeal carcinoma in the training cohort.

Variables	Overall survival			Cancer-specific survival		
	HR	95% CI	*P*	HR	95% CI	*P*
Total						
Age group (years)						
<50	Reference			Reference		
50-64	1.835	1.601-2.103	<0.001^∗^	1.652	1.422-1.920	<0.001^∗^
≥65	3.327	2.866-3.863	<0.001^∗^	2.513	2.120-2.979	<0.001^∗^
Race						
Black	Reference			Reference		
White	0.946	0.809-1.107	0.488	0.879	0.735-1.051	0.158
Asian/Pacific islander	0.737	0.620-0.875	<0.001^∗^	0.781	0.644-0.948	0.012
Indian/Alaska native	1.411	0.978-2.034	0.065	1.399	0.923-2.120	0.114
Marital status						
Single	Reference			Reference		
Married	0.866	0.754-0.993	0.040	0.894	0.765-1.045	0.160
Divorced	1.282	1.065-1.544	0.009	1.279	1.033-1.584	0.024
Windowed/separated	1.406	1.143-1.729	0.001	1.376	1.079-1.753	0.009
Histology						
KSCC	Reference			Reference		
DNKC	0.715	0.619-0.824	<0.001^∗^	0.718	0.611-0.844	<0.001^∗^
UNKC	0.629	0.512-0.775	<0.001^∗^	0.583	0.457-0.742	<0.001^∗^
Others	0.920	0.804-1.054	0.229	0.870	0.745-1.017	0.081
Grade						
Well	Reference			Reference		
Moderate	0.911	0.635-1.306	0.612	0.915	0.599-1.398	0.682
Poor	0.794	0.565-1.116	0.184	0.794	0.532-1.185	0.259
Undifferentiated	0.759	0.529-1.088	0.133	0.735	0.481-1.123	0.154
Unknown	0.819	0.581-1.156	0.257	0.794	0.530-1.189	0.263
Tumor size (cm)						
<3	Reference			Reference		
3-5	1.281	1.084-1.514	0.004	1.268	1.041-1.544	0.018
>5	1.667	1.368-2.031	<0.001^∗^	1.751	1.398-2.194	<0.001^∗^
Unknown	1.482	1.270-1.728	<0.001^∗^	1.523	1.270-1.827	<0.001^∗^
AJCC stage						
I	Reference			Reference		
II	1.378	1.038-1.828	0.026	1.612	1.118-2.324	0.011
III	1.74	1.292-2.344	<0.001^∗^	2.577	1.772-3.747	<0.001^∗^
IV	1.632	1.165-2.287	0.004	2.508	1.668-3.771	<0.001^∗^
T stage						
T1	Reference			Reference		
T2	1.045	0.891-1.225	0.589	1.097	0.912-1.319	0.327
T3	1.218	1.013-1.465	0.036	1.146	0.931-1.411	0.198
T4	1.611	1.281-2.025	<0.001^∗^	1.528	1.193-1.957	<0.001^∗^
N stage						
N0	Reference			Reference		
N1	0.899	0.770-1.051	0.182	0.918	0.767-1.099	0.351
N2	0.925	0.785-1.091	0.356	0.892	0.740-1.076	0.232
N3	1.368	1.089-1.717	0.007	1.34	1.047-1.716	0.020
M stage						
M0	Reference			Reference		
M1	2.926	2.437-3.512	<0.001^∗^	3.278	2.701-3.978	<0.001^∗^
Chemotherapy						
Yes	Reference			Reference		
No/unknown	1.488	1.274-1.738	<0.001^∗^	1.593	1.328-1.911	<0.001^∗^

## Data Availability

If the original data is reasonably required, the permission to obtain the data can be applied to Professor Zhiru Li.
